# Backbone flexibility controls the activity and specificity of a protein-protein interface – specificity in snake venom metalloproteases (SVMPs)

**DOI:** 10.1186/1758-2946-3-S1-O22

**Published:** 2011-04-19

**Authors:** Hannes G Wallnoefer, Torsten Lingott, José María Gutiérrez, Irmgard Merfort, Klaus R Liedl

**Affiliations:** 1 Institute of General, Inorganic and Theoretical Chemistry, Faculty of Chemistry and Pharmacy, University of Innsbruck, Innsbruck, 6020, Austria; 2 Institute of Pharmaceutical Sciences, Department of Pharmaceutical Biology and Biotechnology, University of Freiburg, 79104 Freiburg, Germany; 3 Instituto Clodomiro Picado, Facultad de Microbiología, Universidad de Costa Rica, San José, Costa Rica

## 

Protein-Protein interfaces have crucial functions in many biological processes [1]. The large interaction areas of such interfaces show complex interaction motifs. Even more challenging is the understanding of (multi-)specificity in protein-protein binding. Many proteins can bind several partners to mediate their function [2].

A perfect paradigm to study such multi-specific protein-protein interfaces are snake venom metalloproteases (SVMPs) [3]. Inherently, they bind to a variety of basement membrane proteins of capillaries, hydrolyze them, and induce profuse bleeding. However, despite having a high sequence homology, some SVMPs show a strong hemorrhagic activity, while others are (almost) inactive [4].

Our results indicate that the activity to induce hemorrhage, and thus the capability to bind the potential reaction partners, is related to the backbone flexibility in a certain surface region[4]. A subtle interplay between flexibility and rigidity of two loops seems to be the prerequisite for the proteins to carry out their damaging function. Presumably, a significant alteration in the backbone dynamics makes the difference between SVMPs that induce hemorrhage and the inactive ones.
